# Therapeutic Strategies to Enhance Tumor Antigenicity: Making the Tumor Detectable by the Immune System

**DOI:** 10.3390/biomedicines10081842

**Published:** 2022-07-30

**Authors:** Daniel Meraviglia-Crivelli, Angelina Zheleva, Martin Barainka, Beatriz Moreno, Helena Villanueva, Fernando Pastor

**Affiliations:** 1 Molecular Therapeutics Program, Center for Applied Medical Research, CIMA, University of Navarra, 31008 Pamplona, Spain; dmeraviglia@alumni.unav.es (D.M.-C.); azheleva@unav.es (A.Z.); mbarainkago@unav.es (M.B.); bmbruma@unav.es (B.M.); evillanue@unav.es (H.V.); 2 Instituto de Investigación Sanitaria de Navarra (IDISNA), Recinto de Complejo Hospitalario de Navarra, 31008 Pamplona, Spain

**Keywords:** neoantigens, tumor immunity, cancer immunotherapy

## Abstract

Cancer immunotherapy has revolutionized the oncology field, but many patients still do not respond to current immunotherapy approaches. One of the main challenges in broadening the range of responses to this type of treatment is the limited source of tumor neoantigens. T cells constitute a main line of defense against cancer, and the decisive step to trigger their activation is mediated by antigen recognition. Antigens allow the immune system to differentiate between self and foreign, which constitutes a critical step in recognition of cancer cells and the consequent development or control of the malignancy. One of the keystones to achieving a successful antitumor response is the presence of potent tumor antigens, known as neoantigens. However, tumors develop strategies to evade the immune system and resist current immunotherapies, and many tumors present a low tumor mutation burden limiting the presence of tumor antigenicity. Therefore, new approaches must be taken into consideration to overcome these shortcomings. The possibility of making tumors more antigenic represents a promising front to further improve the success of immunotherapy in cancer. Throughout this review, we explored different state-of-the-art tools to induce the presentation of new tumor antigens by intervening at protein, mRNA or genomic levels in malignant cells.

## 1. Introduction

Cancer is one of the main causes of death in developed countries, along with cardiovascular diseases [1]. Consequently, there is a continuous need to find novel strategies to fight cancer. Immunotherapy has revolutionized the field of cancer therapy in the last years as it has been shown to work in numerous successful clinical trials [2,3,4,5,6] for different types of tumors. Active cancer immunotherapy is aimed at eliciting an endogenous immune response to seek and destroy delectably malignant cells. The efficiency and selectivity of the immune response are basically driven by the presence of tumor antigens. While most current cancer immunotherapy approaches focus on tuning tumor-reactive T-cell functionality, there are still few strategies aimed at enhancing tumor antigenicity.

Immunosurveillance is defined as the ability of the immune system to discriminate between self and foreign [7], which is key for the development of current cancer immunotherapy strategies. It was demonstrated that immune cells are spontaneously capable of controlling tumors in the early stages and even of leading to their rejection [8]. Nevertheless, spontaneous regression of advanced tumors is an extremely rare phenomenon, estimated to occur in 1 out of 60,000–100,000 cancer cases in the world and possibly triggered by some inflammatory stimulus [9].

The first clinical evidence that showed that an inflammatory response could benefit cancer patients occurred in the 19th century, when Fehleisen and Busch, working independently, reported tumor regressions after erysipelas infection [10]. In the 1890s, William Bradley Coley developed the first anti-cancer therapy that modulated the immune system by treating patients with different mixtures of bacterial strains [11]. During the 20th century, critical discoveries emerged for the advancement of cancer immunotherapy, e.g., the existence of T cells and their vital role in the immune response was described by Miller in 1967 [12], and dendritic cells (DC) were identified by Steinmann in 1973 [13] as the cells able to prime T cells, and later natural killer cells were discovered by Kiessling in 1975 [14]. The most important contribution in the past century ended with the Nobel Prize being awarded to Allison and Honjo for their studies, a recognition of the impact of immunotherapy in cancer treatment. James P. Allison worked in the development of an anti-CTLA-4 blocking antibody to counteract tumor-induced immunosuppression [15], and Tasuku Honjo’s work played a critical role in the development of the anti-PD-1 blocking antibody [16]. The blockade of both targets, CTLA-4 and PD-1, currently constitutes the first line of cancer treatment along with the two classical treatments, chemo- and radiotherapy, which indicates the importance of novel discoveries trying to improve the efficacy of immunotherapy.

## 2. The Basis of an Efficient Immune Response in Cancer

T lymphocytes are the main most specific defensive cells of the immune system against cancer cells. In order to combat different malignancies, T cells must recognize the tumor antigens through their cell-specific T-cell receptor (TCR) and expand to mount an efficient immune response against them [17]. TCRs count with a constant and exclusive T-cell specific variable region responsible for recognizing a particular peptide antigen bound to a major histocompatibility complex (MHC) molecule on the antigen-presenting cell (APC) [18]. It was estimated that in humans, the immune system counts approximately 2.5 × 10^7^ different clonal variants, each featuring a different TCR [19]. This complexity cannot be encoded in single different genes due to the enormous genomic size it would require. Therefore, the way the immune system achieves this large variability is through random rearrangements of a limited set of genes, known as V, D and J, that end up building the large repertoire of TCRs [20]. Not all TCRs obtained during this process are valid. During T-cell maturation in the thymus the lymphocytes undergo a quality control process known as T-cell education, divided into two steps: (1) positive selection, in which only lymphocytes that present a TCR featuring the capacity to recognize peptide loaded in the MHC molecules survive; (2) negative selection or central tolerance. During this second phase, those lymphocytes that display TCRs that bind with high affinity to self-antigen peptides presented on MHC are eliminated to avoid an autoimmune reaction. The TCR rearrangement followed by T-cell education allows for the existence of T cells capable of recognizing with high affinity most foreign protein antigens (not expressed during the T-cell education in the thymus) and of avoiding self-antigen recognition [21].

Apart from central tolerance, lymphocytes have other peripheral mechanisms of tolerance to preclude autoimmune events of lymphocytes with partial self-reactivity that might have escaped the thymus education [22,23]. In order to become licensed to eliminate cells that express cognate reactive peptides present in the MHC, T cells need to receive sequential signals of activation. First, the TCR must recognize the peptide antigen in the context of an MHC molecule on the APC surface with high affinity [18,24]. It was shown that strong binder peptides could elicit better responses than poor ones [24]. This initial stimulus is necessary but not sufficient for the activation of the T cell; it requires the co-stimulation signal encompassing various types of receptors, with CD28 as the main one. Lack of co-stimulation signal leads to anergic T cells with dysfunctional phenotype [25,26,27,28,29]. Finally, the third signal stimulus is provided by cytokines released during the inflammatory process. The cytokine exerts an important function in determining the type of T-cell immune response that is elicited [30,31].

After the first exposition of the antigen to the immune system, second and subsequent responses against the same target are normally faster and more intense. This observation is explained by immunologic memory [32]. Immunologic memory occurs as a consequence of lymphocyte differentiation into memory cells during the inflammatory response. These mature cells can survive for years in the individual [33]. Memory cells are the base principle of vaccines, in which the exposure of the immune system to a certain attenuated pathogen or immunogen elicits an initial response, granting the apparition of memory cells, capable of eliciting strong responses in future infections [32]. In the context of cancer, memory cells, a specific type of memory T cells known as tissue-resident memory cells (T_RM_), have a really important role in antitumor immunology. They have been detected in different types of cancers, such as melanoma, lung cancer, urothelial cell carcinoma or endometrial adenocarcinoma [34]. In addition, in epithelial cancers, the presence of TRMs has been considered a good prognosis value with non-recurrent tumors [34].

The first signal by the TCR is critical, determining the initiation, magnitude and memory of the immune response [35]. In the context of cancer, tumor antigens released from dead tumor cells are captured by patrolling DC, and they migrate to proximal lymph nodes to present the antigen-processed peptide to T cells. The T cells that engage with high affinity through the TCR with DC receive the co-stimulation signals and cytokines to become activated and proliferate. These expanded clones with a unique TCR are able to recognize the tumor cells and destroy them [36]. Tumor antigenicity is an essential factor that determines the initiation and effectiveness of the immune response [37]. As we have mentioned, the mechanisms of central tolerance in which T cells recognize self-antigens are eliminated complicate the task of the immune system to eradicate malignant cells, given that most of their antigens might be recognized as self for descending from healthy tissue and sharing a strong similarity with it. One of the major challenges in cancer immunotherapy consists in finding strong specific tumor antigens that can ‘awaken’ the immune system and elicit an effective response against tumors. Tumor antigens can be classified into three groups: (1) Tumor-associated antigens. In this group, we find those native proteins that are usually not expressed at high levels in regular somatic tissues, such as embryonic proteins or germ protein, or proteins expressed in immune privileged organs, such as testis; (2) Antigens that emerge as a consequence of tumor mutations: neoantigens; (3) Antigens of viral origin, such as those that appear from tumors caused by papillomaviruses. In this review, we discussed the most relevant sources of antigens in cancer, highlighting state-of-the-art strategies that focus on developing therapies that can make tumors more antigenic.

## 3. Cancer Mechanisms of Immune Escape

The induction of an effective T-cell immune response is a highly controlled process that undergoes multiple checkpoints to avoid the devastating effect of autoimmune reactions [21,22,29]. As the tumor progresses, it acquires new capabilities, one of which is to exploit these intrinsic immune checkpoints to evade the immune system [38]. Tumor cells can exploit the signals required for T-cell activation to their own benefit. Apart from co-stimulators, another group of receptors with inhibiting function exists. Physiologically, these groups of molecules guarantee the end of the immune response resolving the inflammation once the pathogen is eliminated, thus preventing healthy tissue damage by the effector immune cells [39]. Tumors take advantage of these pathways to inhibit the antitumor immune response. This is the case of CTLA-4 or PD-1, the two most broadly blocked targets in cancer immunotherapy [39]. Cancer cells can also modify the tumor microenvironment (TME) to foster an immunosuppressive environment that favors the production of cytokines such as IL-10 and transforms growth factor β (TGF-β), among others [40,41]. In addition, malignant cells can also recruit immune cells with inhibiting functions, such as Tregs and macrophages [40], in turn boosting tumor survival.

Given the importance of tumor antigens to mount an effector immune response, tumors also develop mechanisms to counteract tumor antigenicity. Alterations in the process of antigen presentation with loss of MHC or other proteins involved in the pathway, such as the transporter associated with antigen processing (TAP), are commonly observed in different types of tumors [42,43].

Tumors may deploy yet another mechanism of adaptation, known as cancer immunoediting, which consists of the selection of immunologically inert tumor variants. In an early stage of tumorigenesis, highly immunogenic malignant cells can be detected by the immune system and, consequently, eliminated. Nevertheless, those tumor variants that manage to survive due to their ability to avoid the immune response reaches a stage of equilibrium, in which the tumor is dormant and clinically undetectable. In the final step of the development of the tumor, resistant cells proliferate and establish detectable tumors that do not express immunodominant antigens [7,8].

## 4. Current Immunotherapy Approaches

Cancer immunotherapy tries to overcome the mechanisms employed by tumor cells to evade the immune response. One of the best-known examples is immune checkpoints, such as CTLA-4 and PD-1, which are exploited by tumor cells. Some of the most successful cancer immunotherapy strategies are, indeed, immune checkpoint blockers. As discussed in the introduction, the first to arrive in the clinic were antibodies targeting CTLA-4 and PD-1. The anti-CTLA-4 antibody, ipilimumab, was used as monotherapy for melanoma [44], although it is now commonly combined with an anti-PD-1 blocking antibody (nivolumab or pembrolizumab). Several clinical trials reported the success of PD-1 blockade as monotherapy or in combinational treatment for different cancer types: melanoma [45], lung cancer [4], hepatocellular carcinoma [46], or esophageal squamous cell carcinoma [47]. Another approach to intervening in the PD-1 axis consists in blocking the PD-1 ligand (PD-L1) with the antibody atezolizumab. This was successfully employed as monotherapy for different types of tumors such as breast, urothelial and gastric cancers [5,48,49] or in combination with other treatments such as chemotherapy [50].

In addition to immune-checkpoint blockade (ICB) therapy, a different strategy focused on engrafting a new immune response is adoptive cell therapy (ACT). This technique, which was pioneered in patients with melanoma [51], initially meant infusing patients with autologous ex vivo expanded tumor-infiltrating T cells (TILs). The lymphocytes administered to the patient home to the tumor niche and destroy the remaining malignant cells. A great advance in the ACT is the Chimeric Antigen Receptor (CAR) T cells, a strategy that has shown ample success in hematologic cancers [2,52,53]. The first CAR was designed to target the CD19 antigen [52]. The patient was treated with CAR-T cells in combination with chemotherapy. A dramatic regression of the patient’s lymphoma was observed along with depletion of all B-cell lineages. After this initial success, other trials were carried out with CD19 CAR-T cells, accomplishing great clinical benefits in patients with recurrent or chemotherapy-resistant blood cancers with no effective therapies [54,55,56]. The efficacy of CAR-T therapy in metastatic solid tumors is, however, still poor [2]. One of the main limitations to the success of CAR-T therapy in solid tumors is the low abundance of good targetable molecules expressed on the surface of the malignant cells. In an attempt to improve ACT therapy in solid tumors, Tran and colleagues were able to stimulate ex vivo TILs capable of recognizing a specific antigen derived from a KRAS mutation [57]. The resultant reactive lymphocytes were implemented in a clinical trial with a patient with metastatic colorectal carcinoma. After ACT therapy, a clear regression was detected in the lung metastasis, showing the relevance of tumor-specific antigens.

Another strategy that explored focusing the immune response on malignant cells is the development of a cancer vaccine. One of the first cancer vaccines ever reported was GVAX. GVAX was based on inactivated-by-radiation tumor cells, avoiding their proliferation which might lead to new tumors in the patient; moreover, they are engineered to express and secrete granulocyte-macrophage colony-stimulating factor (GM-CSF). This vaccination, designed for the first time in the melanoma mouse model B16, elicits a strong T-cell-dependent antitumor immune response in animals [58]. More recently, some clinical trials were carried out to bring GVAX into the clinic. Despite the promising results of GVAX detected in mice, clinical trials have not been so successful thus far. This is a technically cumbersome approach. The best GVAX formulation requires the generation of a specific cell line derived from each patient’s tumor sample to genetically modify it to express GM-CSF. Simpler approaches aimed to develop an off-the-shelf vaccine using modified allogeneic cell lines did not work out, probably because they neglected the importance of tumor-specific antigens in the effectiveness of the antitumor immune response [59]. Tumor neoantigens are probably the strongest kind of antigens that determine the outcome of the antitumor immune response. They are mainly derived from mutations acquired as the tumor progresses. The value of tumor neoantigens hinges on the foreign immunological identity of these kinds of proteins since they are not encoded in the genome and thus are not subjected to central immunological tolerance, leading to immune responses driven by lymphocytes with high-affinity TCR engagers. This is so important that, in many instances, tumor mutation load, which is proportional to the number of potential neoantigens, was underscored as a positive predicted value in treatment with ICB [60]. Recent work tried to shed light on antigen discovery as an attempt to develop personalized neoantigen vaccines. This type of vaccine requires the prediction of tumor-specific antigens to foster the anti-cancer immune response. First, mutations present only in malignant cells are identified by full-exome sequencing of tumor and healthy germ line tissue. Then, the expression of mutated transcripts by the malignant cells is verified by RNAseq [61]. Finally, a peptide MHC-binding algorithm allows for the prediction of potential neoantigens in conjunction with mass spectrometry to identify MHC-bound peptides. Ott and colleagues predicted and validated up to 20 patient-specific tumor neoantigens using this vaccination approach. Furthermore, the authors reported that four out of ten melanoma patients enrolled in the study remained disease recurrence-free; a further two patients, after anti-PD-1 ICB therapy, showed complete responses [62]. Cancer peptide vaccines can be administered with adjuvants that enhance the immune response, such as the TLR-3 and MDA5 agonist poly-ICLC employed in NeoVax. Personalized cancer vaccines are being used in ongoing different clinical trials in combination with ICB therapy in several types of tumors, such as ovarian carcinoma, renal carcinoma, glioblastoma, or lymphocytic leukemia (NCT04024878, NCT02950766, NCT02287428, NCT03219450). In addition to peptides as a source of antigens, mRNAs encoding for tumor neoantigens can also be used. The mRNA-based vaccines are administered as nanoplexes with different formulations intradermically or directly injected into the lymph node to favor T-cell priming. Sahin et al. successfully employed the latter strategy in melanoma patients [63]. Several personalized mRNA vaccines are currently being evaluated in clinical trials for different types of cancer, such as breast, melanoma, renal or colorectal cancers (NCT00003432, NCT03480152, NCT00087984, NCT00003433). A recent review of the current state-of-the-art in personalized cancer vaccines with detailed information on clinical trials was published by Liu et al. [64]. Despite the promising results, personalized cancer vaccines present some important hurdles. Because they constitute a patient-specific therapy, this requires a specific antigenic prediction study for each case, which is both costly and technically cumbersome [61], as RNAseq and full exon sequencing from the tumor and healthy tissue are required. Moreover, the identification of mutations by sequencing drags the limitations along. Methods tend to rely on assembling the sequence of interest by aligning short reads, which often fails to identify indel frameshift mutations [65,66]. However, another limitation of cancer vaccines is tumor heterogenicity and the imperative need to identify clonal mutation-derived neoantigens to avoid tumor escape. Current vaccines rely on a limited number of epitopes that might target only a small fraction of cancer cells [61]. Finally, personalized vaccines rely on the tumor mutational burden (TMB) and the antigen-presenting machinery of the tumor, reducing their effectiveness in tumors with a low TMB or alterations in the antigen presentation machinery.

When considering the key aspects of tumor antigenicity in cancer immunotherapy, therapeutic interventions aimed at increasing the quality and quantity of tumor antigens have been highly pursued over the last few years. These types of approaches can be summarized depending on where the tumor cells are targeted: (i) alteration in antigen presentation pathways; (ii) mRNA maturation and turnover; (iii) unleashing genomic cryptic elements; (iv) expression of exogenous protein antigens.

## 5. Main Approaches to Enhance Tumor Antigenicity

### 5.1. Alteration in Antigen Presentation Pathways to Elicit Tumor Antigens

**Figure 1:**
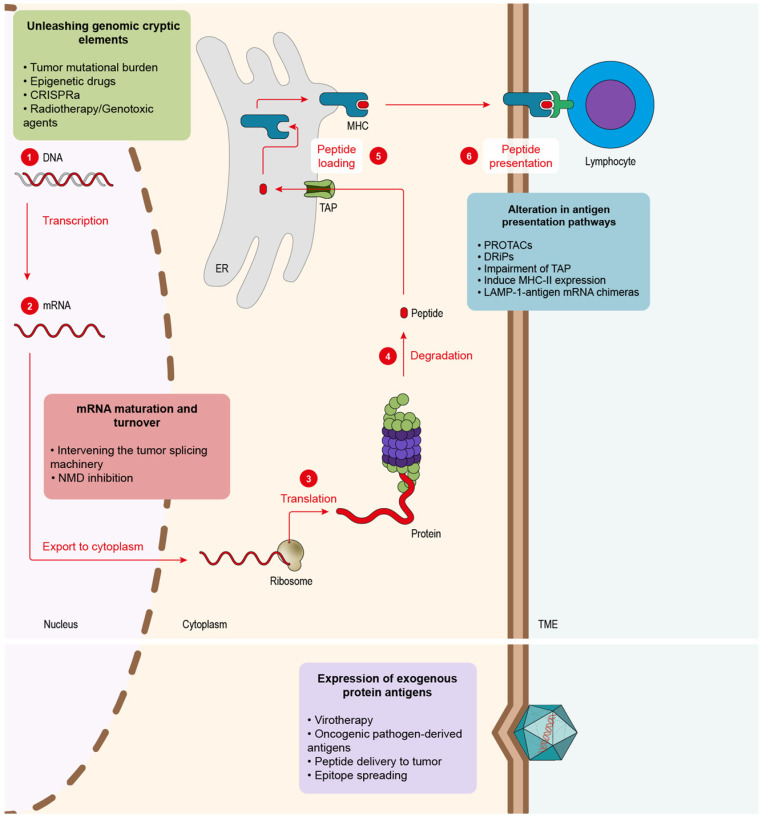
Tumor antigenicity can be enhanced at different levels. Endogenous tumor antigens can be fostered by altering different pathways in the tumor cells: 1. Genomic DNA contains an excellent source of dormant antigens that can be unleashed via several strategies: epigenetic drugs, radiotherapy, genotoxic drugs or CRISPRa. 2. Modulation of mRNAs maturation and homeostasis can lead to novel tumor antigens. Aberrant mRNAs might be generated via splicing inhibition or NMD blockade. 3–6. Proteins must be degraded to obtain antigenic peptides that are refined in the ER until the final ones are presented via MHC. Several strategies can be used to boost peptide-derived neoantigens at the protein level: PROTACs that induce selective target protein degradation, approaches that favor DRiP generation, TAP inhibition, induction of MHC-II presentation in tumor cells. Another source of antigens can be supplied by exogenous proteins.

CD8 T cells recognizing peptide antigens presented on the tumor cell surface are bound to MHC-I molecules. The main source of MHC-I peptides is obtained through the protein degradation that takes place in the proteasome, generally in a ubiquitin-dependent manner. After proteolysis, the resultant peptides are imported into the endoplasmic reticulum (ER) via the transporter TAP and finally loaded into the MHC-I that presents the antigens on the cell surface so that CD8 T cells can scan and recognize them (Figure 1). Given the importance of the ubiquitin pathway in the processing of MHC-I peptides, once a strong antigen has been characterized, and its source is known, it would be interesting to boost the degradation of the original protein in order to enhance the peptide presentation in cancer cells. This is what proteolysis targeting chimeras (PROTACs) do. PROTACs are small molecules that target a specific protein to proteasomal degradation by recruiting ubiquitin E3 ligases to knock down protein activity for therapeutic purposes. In order to achieve it, PROTACs have two functional cores, one acting as a ligand of the target protein and another recruiting the ubiquitin E3 ligases. Jensen and colleagues [67] tested three different PROTACs and were able to track the resultant peptides in the immunopeptidome of the treated cells. Massafra and colleagues [68], on the other hand, demonstrated that the peptide presentation induced with PROTAC treatment could activate T cells against human cancer cell lines in vitro. These types of drugs could be used to elicit an immune response against common tumor antigens (e.g., tumor oncogenes).

We have mentioned that the key transporter of peptides into the ER is TAP, a heterodimer formed by TAP1 and TAP2 subunits. The inhibition of this transporter partially inhibits the canonical peptide presentation, thus favoring an alternative source of peptides [69]. Hence, the impairment of TAP function in tumor cells offers two main advantages: (1) it triggers the presentation of peptides by the non-canonical pathway and thus makes them highly antigenic since they are not present in normal cells [70]; (2) these non-TAP dependent antigens might be mutation-independent, which makes them more prone to be shared across all tumor types [70,71]. In order to inhibit TAP, specifically in cancer cells, Garrido and colleagues [72] designed an aptamer-siRNA chimera that was able to target the malignant cells and trigger the impairment of TAP. Their results showed an increase in T-cell infiltration followed by a decrease in tumor growth in mice and better overall survival. Finally, TAP inhibition presented an additive effect with ICB therapy (anti-PD-1 antibody).

Apart from the MHC-I-dependent peptides, which are recognized by CD8 T cells, APCs also count, with MHC-II being recognized by CD4 helper lymphocytes. CD4 T lymphocytes play an important role in assisting the antitumor immune response [73]. MHC-II expression in tumor cells was reported in different cancer types, such as melanoma [74], in which it correlated with better clinical outcomes and response to PD-1/PD-L1 ICB therapy or breast cancer [75], and is also associated with better prognosis and higher immune infiltration. Some studies performed in mice exploring the induction of MHC-II expression in cancer cells presented promising results in breast cancer models [76].

Another way to boost MHC-II-dependent antigen presentation consists in delivering the antigen through the lysosomal pathway, such as the lysosome-associated membrane proteins (LAMPs). In order to achieve this, LAMP-1 and the antigen’s mRNA are fused to generate a chimeric mRNA that is then transfected into DCs. This approach was tested with carcinoembryonic antigen (CEA). Researchers observed the expansion of CEA-specific CD4^+^ T cells, which led to a stronger CD8 T-cell cytotoxic response to the same antigen in vitro [77]. The concept was tested in different phase-I clinical trials in glioblastoma patients (NCT02529072, NCT00626483, NCT00639639), showing an improvement in the overall survival of patients. In addition, the treatment is ongoing through several phase-II clinical trials trying to improve efficacy via combination with other drugs, such as antagonistic antibodies, GM-CSFG or tetanus toxoid preconditioning to enhance DC migration (NCT02366728, NCT03927222, NCT03688178, NCT02465268).

We discussed the peptides originated from the degradation of full proteins and how they can be tuned to enhance antigen presentation. However, another source that would likely lead to fast-presented peptides is defective ribosomal products (DRiPs). The DRiP hypothesis suggests that peptides could emerge from translation products that cannot, or do not, achieve a stable structure and are rapidly degraded [78,79]. The existence of DRiPs was studied mostly in viruses. Some studies proved that MHC-I peptides derived from viral products could be detected before the expression of the viral proteins per se [80,81]. In tumors, a work from Yewdell’s group showed that the knockdown of certain ribosomal proteins could boost the externalization of some MHC-I haplotypes without changing the total expression of MHC-I in the cell. This study also points to the fact that targeting ribosomes in cancer cells to change the antigen repertoire might help the immune system detect them as well as elicit strong antitumor responses [82].

### 5.2. Alteration of mRNA Maturation and Turnover to Foster New Tumor Antigens

Another strategy to induce the emergence of neoantigens is exploiting mRNA maturation and homeostasis (Figure 1). Errors during mRNA maturation may lead to aberrant transcripts that might, in turn, codify for potential neoantigens. Impaired expression of splicing modulators across several cancer types was detected, as in the case of U2AF1 and SF3B1, which were found mutated in myelodysplasia and chronic lymphocytic leukemia [83,84]. Additionally, a study performed on lung adenocarcinoma discovered that splicing impairment in cancer cells triggers the presentation of a new plethora of neoantigens derived from aberrant transcripts [85]. They also were able to identify the novel peptides and validate them in humanized mice. In order to exploit these splicing-originated neoantigens, some studies tested different antitumor therapies using splicing inhibitors. In 2021, Lu and colleagues [86] implemented two different splicing inhibitors, Indisulam and MS-023, showing that they could elicit a potent antitumor response mediated by neoantigens, which they identified while analyzing the immunopeptidome. They also demonstrated that peptides could be used to vaccinate mice and induce the activation of the immune system against the tumor cell in vitro. This strategy showed significant synergy in combination with PD-1 ICB therapy. Closer to the clinic is the splicing inhibitor E7820, which is currently in a phase-II trial (NCT05024994) in patients with myeloid cancers that show mutations in splicing factors. Apart from the neoantigen splicing inhibition awakening, the aberrant splicing machinery that many tumor cells harbor can offer further promising therapeutical opportunities that may complement the boost of neoantigen presentation. North and colleagues [87] showed that it was possible to deliver a well-characterized druggable target protein as an unprocessed mRNA that could be only spliced by the malignant cells. In this work, the authors employed cells that presented a mutated form of the splicing factor SF3B1. Cells were transduced with herpes simplex virus–thymidine kinase (HSV–TK) mRNA containing synthetic introns that could only be spliced by cells containing aberrant SF3B1, leaving healthy tissues untouched. In this way, only tumor cells were capable of processing the HSV-TK transcript, which conferred them sensibility to ganciclovir, an FDA-approved compound for herpes simplex [87].

Cancer cells can harbor multiple mutations in their genomes that result in the transcription of aberrant mRNAs. Nevertheless, not all mutations have the same immunogenic potential. In order to address this issue, the mutation type needs to be considered. According to its origin, we may find two kinds of mutation: (1) single-point mutations and (2) frameshift mutations. (1) Single-point mutations cause only one nucleotide substitution in the DNA, which leads, in the best-case scenario, to a new amino acid in the peptide sequence. (2) Frameshift mutations, on the other hand, trigger the formation of a novel sequence, opening the possibility of a new array of neoantigens radically different from the initial protein [60]. Despite their antigenic potential, many frameshift mutations lead to the creation of premature stop codons (PTCs). PTCs in messenger RNA (mRNA) are identified by the nonsense-mediated decay (NMD) machinery and degraded, so the potential antigens coded by this aberrant transcript are lost. It was observed that some PTC-containing mRNAs manage to escape NMD, supporting the importance of these NMD-dependent antigens in the clinic. In these cases, patients show a significantly better response to ICB therapy [88,89]. A strategy to recover the remaining peptides that NMD suppresses consists of compromising NMD activity. Pastor et al. (2010) [90] showed that NMD inhibition leads to tumor immunity by possible stabilization and presentation of this type of neoepitopes. In addition, recent work reported that NMD inhibition via an aptamer-siRNA chimera induced a boost in immune infiltrate and slowed tumor growth [91,92]. Another evidence of the importance of NMD in cancer was observed in mismatch repair (MMR) deficient colorectal cancer (CRC) with microsatellite instability (MSI). These types of tumors accumulate a high number of mutations due to their poor DNA repair capacity. It was observed that CRC MSI^+^ tumors display high NMD activity, probably to cope with the substantial levels of aberrant transcripts from the mutant genes. In fact, NMD inhibition was found to present a deleterious effect, slowing cell growth in vitro and tumor growth [93].

### 5.3. Unleashing Genomic Cryptic Elements

We reviewed the principal sources of antigens at mRNA and their relevance in cancer immunotherapy. mRNAs, however, represent only a small fraction of the genome that is actively transcribed. Targeting the genome of cancer cells could trigger a vast array of novel antigenic peptides [94]. The main sources of neoantigens comprised in the genome originated from mutations present in malignant cells, which they acquire during the process of tumorigenesis (Figure 1). The presence of mutations, also known as TMB, was determined as a key biomarker in cancer. Clinical trials in several cancer types, such as lung [4] and melanoma [95], showed that high TMB correlated with better prognosis in combination with different ICB immunotherapies. One special case is tumors with microsatellite instability caused by a deficiency in MMR. In normal cells, MMR is a housekeeping mechanism that corrects base-to-base mismatches produced by exposure to DNA damage mediated by exogenous chemicals or physical agents (e.g., cigarette smoke) as well as some endogenous reactive metabolites (e.g., oxygen reactive species). In tumors that lack MMR, DNA accumulates a high number of somatic mutations, leading to the production of neoantigens associated with these mutations [96]. The presence of this potent immune response leads to boosting ICB treatment outcomes [6,97] in these tumors with MMR.

Genomes present multiple dormant elements, such as mobile elements, viruses and mutated gene copies [98] that could be able to originate different neoantigens. Epigenetic machinery executes vital regulatory processes that regulate the expression or repression of many elements in the cell genome [99]. Intervening in epigenetic events to activate genes involved in the MHC presentation pathway, immune-checkpoint genes such as PD-L1 (which can be targeted with ICB therapies or trigger the emergence of neoantigens) hold vast potential for the development of novel strategies to make tumors more antigenic. One of the epigenetic processes that can be targeted is DNA methylation, which is involved in gene repression and catalyzed by DNA methyltransferases (DNMTs). DNA methylation inhibitors, such as 5-aza-2-deoxycytidine (DAC), were FDA-approved for the treatment of hematological malignancies [100]. Preclinical data from this study showed that DAC might induce the demethylation of aberrant CpG islands producing dsRNAs that end up in the upregulation of type-III interferons (IFN). However, the mechanism of efficacy of this compound remains controversial. In addition to global demethylation, some studies point to DAC leading to the inhibition of NMD [101], which, as discussed earlier in this review, triggers the stabilization of aberrant mRNAs leading to the emergence of a potential array of neoantigens. Recently, a novel study showed that treatment with DAC in glioblastoma, a tumor with a really low TMB, induces the presentation of MHC-I-dependent neoantigens [102], demonstrating the high potential of epigenetic drugs in cancers with low mutational levels. The researchers treated patient-derived glioblastoma cells with DAC and co-cultured them with tumor-infiltrating lymphocytes (TILs) isolated from patients ex vivo, showing a significant immune reactivity boost when tumor cells were treated with DAC.

In addition, a phase-II clinical trial with guadecitabine, a decitabine analog, showed promising clinical results in patients with peripheral T-cell lymphoma. Guadecitabine upregulated proinflammatory signaling pathways (e.g., type I and II interferons, TNF-α and the JAK/STAT pathways). Moreover, genes associated with antigen presentation were also upregulated (e.g., β-2-microglobulin, TAP1) [103].

Similarly, another strategy to ‘awaken’ antigens consists in employing a variant of CRISPR/Cas technology called CRISPR activation (CRISPRa). CRISPRa comprises a catalytically dead Cas9 (dCas9) but keeps its ability to bind to genomic DNA via recognition of the small guide RNA (sgRNA) while engineered with transcription activators. Thus, after the docking of Cas9 to the target gene, its expression is triggered [104]. In the context of tumor antigenicity, Wang and colleagues showed that the activation of different genes through a multiplexed library of sgRNAs induced the presentation of antigens by cancer cells and elicited a potent antitumor immune response in mice [105].

Not all tumors present a high TMB that can be targeted to induce the presentation of neoantigens. Classic tumor therapies such as radiotherapy or chemotherapy drugs, such as cisplatin, present a strong genotoxic effect on DNA, causing lethal mutations that lead to cell death. Interestingly, the property of these treatments to induce mutations shows a clear potential in turning tumors more antigenic. There is some evidence of radiotherapy enhancing antitumor immune response by upregulating the expression of mutated genes leading to the presentation of neoantigens [106]. In this work, the authors managed to identify MHC-I and MHC-II-dependent peptides induced by radiotherapy treatment. Vaccination with a combination of these antigens, moreover, slowed tumor growth. In the clinic, radiotherapy has shown that it can dramatically improve the therapeutical outcome of ICB in metastatic cancers [107], which the authors hypothesize might be due to the antigen-presentation boost radiotherapy induces.

### 5.4. Expression of Exogenous Protein Antigens

mRNA homeostasis in cancer cells has proved to offer different strategies to promote tumor immunity, such as NMD or splicing modulation. Genomic mutations constitute a further important source of neoantigens that can trigger the immunogenicity of malignant cells. However, as reviewed here, not all tumors present a high TMB. Epigenetic drugs and splicing modulators might cause certain side effects such as short-term memory loss, thrombocytopenia, anorexia, fatigue, bleeding, anemia or joint pain [108,109]. A potential therapeutical approach to overcome this limitation is oncolytic viruses (Figure 1). Some strains present the ability to infect malignant cells preferentially [110], which makes them an interesting tool for developing therapies. Additionally, viruses can be modified in order to redirect their tropism towards cancer cells [110]. Another advantage of these pathogens is that they are detected as foreign elements by the immune system, which triggers strong immune responses [110]. Viral proteins and nucleic acids can serve as a source of neoantigens if presented specifically in tumor cells [111]. This is quite relevant for tumors that lack a high TMB and show low T-cell infiltration levels. Friedman and colleagues (2021) published a clinical trial demonstrating that the treatment with the oncolytic virus G207, engineered from the herpes simplex virus type 1 (HSV-1), which was capable of increasing the immune infiltrate of the tumors improving the patient’s survival [112]. Viruses were also widely engineered as vectors to express proinflammatory proteins [113]. Preclinical data from experiments performed in mice showed that the oncolytic virus Delta-24-RGD could be engineered to express costimulatory ligands, such as 4-1BBL or OX40L, in the surface of malignant cells, increasing tumor infiltration [114,115]. In this line of work, oncolytic viruses can be modified to express cytokines. The most successful approach to this kind of strategy is Talimogene laherparepvec (T-VEC), based on herpes simplex type-1 derived oncolytic virus, designed to replicate specifically in malignant cells and to express GM-CSF. During a clinical trial in advanced melanoma, T-VEC therapy showed a significant improvement in overall survival in the patients [116].

Similarly, a virus that does not show oncolytic properties can serve as a source of antigens. The influenza virus is also capable of infecting both tumor cells and healthy ones. The presentation of influenza viral antigens in both cases turns the immune system towards the malignant tissue, showing a significant improvement in mice with lung cancer as well as an additive effect with an anti-PD-1 antibody [117].

Despite their therapeutical benefits as vectors, it was observed that, in some situations, previous infections caused by some viruses that express pro-oncogenic proteins contribute to cancer development [118]. These are the cases of Epstein–Barr Virus (EBV), a type-4 herpes virus that has been related to Burkitt lymphoma, or Papillomavirus involvement in Barrett’s dysplasia and esophageal adenocarcinoma [119]. In some cases, during the infection, some parts of the viral genome can be integrated into the genome of the infected cell. This could lead to the expression of viral exogenous proteins that can be presented via MHC-I in the tumor, serving as tumor-specific ones, which would allow targeting malignant cells and developing or repurposing existent vaccines to treat or prevent tumor growth. Recent research [120] showed that an EBV vaccine based on the implementation of four viral glycoprotein-decorating nanoparticles tested in mice could block the viral infection. Additionally, the animals did not develop any lymphomas associated with the virus.

In addition to viruses, another method to address the lack of endogenous antigens in the tumor could be the delivery of MHC-I peptides to tumor cells (Figure 1). During the peptide binding to MHC in the ER, the peptide editor, known as tapasin-related protein (TAPBPR), is essential to shape the final presented peptide [121]. Interestingly, TAPBR has shown the ability to decorate tumor cells with different exogenous peptides present in very low concentrations and to trigger CD8 cytotoxic responses, a promising strategy to make tumors more antigenic [122].

In line with this work, Kavunja and colleagues [123] designed another tool that allowed for the delivery of known MHC-I-dependent antigens to the TME, aiming to elicit an antitumor CD8 response. The authors designed a microparticle capable of delivering peptides in vivo to the tumor and releasing them by degradation in the low pH of the TME. They validated their approach using the ovalbumin-derived peptide SIINFEKL: mice were vaccinated with ovalbumin prior to the inoculation of the tumors, which did not express the protein nor the peptide endogenously. The delivery of the microparticles containing SIINFEKL showed the induction of a potent CD8 immune response which significantly improved the survival of the treated mice [123].

As reviewed in the previous sections, cancer immunotherapy focuses on exploiting the immune system’s endogenous mechanisms to improve its response to malignancies. Once the immune response is unleashed by one of the aforementioned therapeutic interventions, the immune response can expand to other tumor antigens due to a phenomenon known as epitope spreading [124]. This process acts as a cascade expanding the array of antigens that the immune system employs to target the tumor: lysis of cancer cells by CD8^+^ T lymphocytes through the recognition of an initial peptide permits DCs to obtain access to the dead cells’ cytosolic components. DCs then process and present the new epitopes, which can activate new clones of CD8^+^ and CD4^+^ lymphocytes through MHC-I and MHC-II antigenic presentation [125]. The relevance of this phenomenon for cancer was described in mice and some vaccine clinical trials. In animal models, vaccination with ovalbumin (OVA)-expressing cells pulsed with the OVA-derived peptide SIINFEKL induced not only CD8 responses to the initial peptide but to other OVA-derived peptides. Mice thus immunized reject tumors [126], showing the importance of epitope spreading in tumor immunology. Patient data from a phase-I clinical trial in which renal carcinoma patients were vaccinated with DCs loaded with MHC-I and MHC-II-dependent peptides showed a positive response to therapy; of note, the vaccine-induced T-cell responses against other targets that were not included therein. This final observation indicates that epitope spreading may occur, boosting the clinical outcome of DC vaccinations [127].

## 6. Summary

T cells constitute the first line of defense against cancer; the decisive step to trigger their activation is mediated by antigen recognition. Despite being derived from self-tissues, tumors are subjected to immune recognition. As the tumor evolves, acquiring new capabilities to survive and propagate, mutations in the genome of malignant cells are accumulated. All these mutations become a potential source of neoantigens that can elicit an antitumor response; depending on the TMB, each tumor might be more or less prone to immunotherapy interventions [60].

There is a promising horizon in the development of personalized vaccines that consists of the identification of these unique mutations in malignant cells using next-generation sequencing and MHC-predictive algorythms in conjunction with mass spectrometry to decipher the peptide MHC ligandome. This is a technically cumbersome approach, far from accessible to most patients, which still requires further optimization for the discovery of relevant immunodominant clonal neoantigens.

The tumor develops mechanisms of immune evasion and tumor immunoediting. Malignant cells are selected to escape potent immunodominant antigen expression and counteract immune responses. Furthermore, many tumors display considerably low TMB. Therefore, alternative feasible therapeutic strategies are required so as to maintain and/or renew tumor antigenicity. We reviewed and suggested different approaches to achieve this goal: (i) altering the genome and epigenomic landscape; (ii) interfering with mRNA maturation and pathways of mRNA decay; (iii) altering the canonical antigen presentation pathway and the rate of protein turnover; (iv) expressing exogenous antigens.
